# Atlanta Society of Medicine

**Published:** 1890-07

**Authors:** 


### ATLANTA SOCIETY OF MEDICINE.

Vice-President Dr. F. W. McRae in the chair. Regular meeting for reports of cases, June 3, 1890. Dr. Gaston reported a case of urethrotomy without subsequent dilatation.

### DIVISION OF STRICTURE OF THE URETHRA WITHOUT SUBSEQUENT SOUNDING OR DILATATION.

On the call for reports of cases, the following remarks were made by Dr. J. McF. Gaston :

Having on a previous occasion reported a case of aspiration of the bladder and subsequent external urethrotomy for complete occlusion of the urethra, a further history of the case will be likely to interest the society.

Being called to the same patient in April, he was found suffering from urinary infiltration into the scrotum and adjacent tissues, with great constitutional disturbance. Notwithstanding the continuation of free flow of urine through the perineal opening, the urine had been forced into the abscess cavity in advance of it, and not having an outlet anteriorly through the penis, it penetrated into the cellular structures by infiltration, producing its usual results. He was ordered internally the following : ℞. Nitrate potash, ʒi; calomel, grs. vi.; tartar emetic gr. i.; divided in six powders; one to be taken every two hours. An external application was made as follows : ℞. Mercurial ointment, belladona ointment *aa* ℥ss ; pulv. camphor ʒi ; mix and smear over the scrotum and neighboring parts with cotton and a supporting bandage.

Some relief being afforded, the patient returned shortly afterwards to the Providence Infirmary, where a free outlet through the fistulous opening was secured by an incision, and ultimately it was found that a small amount of urine could be forced through the penile urethra. At this stage Dr. Elkin succeeded in passing a filiform whalebone bougie through the stricture into the bladder.

By careful manipulation with the staff-loop of Massonneuve's lithotome, it was guided into the bladder, and the blade was used to divide the stricture up to the point of admitting a No. 32 sound with facility.

It was thought best in view of all the past history of this case not to follow up with the use of the sound and this operation being done on May 1st there was no repetition of the sounding or other measure for dilatation until the 25th of the month. The urine had been passing freely during this period by the perineal fistula and by the urethra, but it was deemed expedient to verify the caliber of the urethra, preparatory to discharging the patient from the Infirmary.

A 4 per cent. solution of cocaine was injected into the canal, and upon introducing a No. 12 English catheter, it was arrested a little in advance of the perineal fistula. It seemed to me that it might have lodged in a pocket resulting from the former abscess of this part and it was suggested to Dr. Elkins to pass a large female catheter through the fistulous opening forward, which was passed readily until it came in contact with the catheter in the perineal urethra. By withdrawing the male catheter until the end of it rested upon the end of the female catheter, and then urging it forward along the line taken by it, there was no further difficulty in reaching the bladder. A No. 26 sound was then introduced, and subsequently a No. 30 passed without difficulty, indicating a calibre entirely satisfactory, so that the patient may now be discharged.

The practical question as to the utility of passing the sound for the purpose of preventing the union of the margins of a divided stricture, is well presented in this case. It affords conditions likely to satisfy impartial observers that when a contracted elastic band forming a stricture of the urethra is completely divided at one point, there is no necessity for dilatation by bougie or sounds. If the incision passes entirely through all the fibers of this ring, it is evident that the elasticity of the tissues must draw the edges apart and any means of dilatation which may be subsequently employed acts upon the intervening cellular tissue without distending the proper components of the gaping indurated stricture. We should naturally expect the gradual absorption of the components of the divided band if left at rest, whereas by the irritations of bougies or sounds this process would be materially retarded, if not prevented.

Under the very unfavorable conditions of this case it is found that after the lapse of twenty-five days, without the introduction of a sound or bougie the calibre of the urethra is preserved so as to afford a full flow of urine; and the result warrants the repetition of this procedure by those who may have to operate on close strictures of the urethra.

In discussing Dr. Gaston's paper, Dr. Todd said while he had never resorted to that plan of treatment for deep strictures, he had observed that subsequent dilatation was not necessary after incision of the meatus, and that he saw no reason why the same thing should not hold good in the deep urethra. Dr. Cooper said he would be very glad to believe that complete division of the stricture was all that is necessary, but that he had always been afraid to adopt that plan of treatment. He would be very glad to avoid subsequent introduction of instruments both for the patient's sake and his own. He said he was then treating a patient from Kansas City, who told him that he had been previously operated upon by a surgeon who did not use any instruments after the operation. The stricture, he said, was one of the tightest strictures he had ever had to deal with, and it was only after repeated efforts that he was able to introduce a filiform.

Dr. McRae said his experience did not tally with Dr. Todd's, and that in one or two instances where he had not kept the incision open he had found it necessary to operate a second time. Theoretically, he was inclined to differ with Dr. Gaston, as it seemed to him that it was necessary to keep the cut open so that it might fill up from the bottom, instead of a direct growing together, which it seemed to him was very apt to take place if bougies were not used after the operation to prevent that growing together.

Dr. Todd then reported several cases illustrating the necessity for more frequent and careful examinations of the urine. One of the cases was of especial interest on account of its subsequent history. The patient was a man who had passed through the hands of a number of physicians, and he only gave a history of indigestion and general bad feeling. An examination of the urine revealed only a trace of albumen, but hyaline and granular casts. The patient died suddenly the following morning, perhaps from heart failure.

Dr. Baird, upon call, reported the case of a young lady recently married, which had given him considerable anxiety for several days. It proved to be merely remarkable inactivity of the bowels, but he feared at the time, notwithstanding the absence of grave, local or general symptoms, that it might be intestinal obstruction of a serious nature.

The facts are briefly these: After several days of unnatural freedom of the bowels, the stools showing imperfect digestion, two days passed without any action. On Tuesday night she took, on her own motion, five grains of calomel, no result following. On Wednesday night she took two grains more without effect. Thursday she took a seidlitz-powder and Thursday night a large dose of castor oil, with negative results. Friday, becoming alarmed, she consulted him.

Found no fever, no pain, tenderness or tympanites. Tongue moist and clean, pulse natural and no nausea or vomiting. Ordered restricted and liquid diet. A seidlitz-powder and a bottle of the solution of citrate of magnesia during the afternoon, to be repeated, if necessary, next morning and to be followed, unless the evacuation should be copious by a simple enema. To his surprise, he found on Saturday, notwithstanding his instructions had been faithfully followed, and in addition, that the patient had voluntarily taken several cathartic pills, no movement had been secured. He then directed that three ounces of castor oil be thrown into the rectum and repeated in about four hours, to be followed next morning by a copious warm water enema.

Sunday, general condition unchanged—no fecal evacuation. Enema returned, showing the presence of some oil but no fecal matter. Examination by the vagina and by the rectum showed no fecal impaction. He then, after consulting with Dr. Armstrong, introduced a long tube into the bowel, as high as the transverse colon, and by means of a Davidson's syringe attached injected a large quantity of warm water, to which some turpentine soap had been added. The injection was continued until the colon was distended and some pain was felt. The water was immediately expelled, deeply stained and having a decided fecal odor, and bringing away two small fecal lumps. The process was at once repeated with similar results, but without the scybalous masses.

Monday.—Patient comfortable, but a little nervous and depressed in spirit, as no movement had occurred. Re-introduced the long tube and injected one quart of olive oil. Directed that it be retained until next morning, when a rectal injection of warm water was to be used. Tuesday.—No fecal movement. General and local condition about the same. Introduced the long tube as before, and threw into the transverse colon eight ounces of castor oil and two drachms of oil of turpentine. Ordered a tablespoonful of castor oil, by the mouth, every two hours until four doses were taken. After about sixteen hours there occurred a fairly free evacuation of fecal matter of soup-like and soft, pasty consistency.

Since then the patient has had daily stools under the influence of mild laxatives.

She was habitually constipated. The rectum was dilated and pouch-like. The injection, without the long tube, did not apparently, go beyond the sigmoid flexure.

Dr. Baird referred to the prevailing fashion of ascribing death to "heart failure." He protested against the use of the term and asserted that it meant nothing. It was on a par with such discreditable expressions as "kidney disease," "liver trouble," "natural causes," etc. With equal propriety might death be said to result from lung failure or brain failure.

The heart in death, of course, fails and that far the expression is true, but it is unscientific, inexact and without qualification, is unintelligible. It conveys no real idea as to the cause of death. It is worthless to the statistician and should not be used in making out death certificates. In some cities, certificates giv- heart failure as the cause of death are rejected by the health department. It is considered equivalent to certifying that the cause is withheld. It would be better and more creditable to the doctor to say that it is unknown. What causes the heart failure ? That is the cause of death.

He appealed to the physicians present to use their influence towards discouraging the use, in . death certificates, of such terms as "heart failure" and "blood poison."

Dr. Virgil O. Hardon reported a case of Batty's operation, and exhibited the specimens. The patient, an unmarried woman about twenty years of age, had suffered for three years with severe and persistent pains in the pelvis, which had undermined her health to such an extent, that of late years she had become bedridden. All other means of cure having been exhausted without effect, Battey's operation was undertaken as a last resort. The left ovary was found to contain a small cyst, while the right one was contracted and cirrhotic. The patient made a good recovery, her temperature at no time exceeding 101. It is yet too early to report the ultimate effects of the operation.

Dr. Cooper reported a case of stricture in which he cut five distinct bands of stricture in penile portion of urethra, and dilated a very tight deep stricture without the patient suffering any untoward symptoms. The points of especial interest in the case were the unusual number and tightness of the strictures and the very rapid recovery.

Dr. Huzza said the case of Battey's operation reported at last meeting had made an uninterrupted recovery. He also mentioned several cases of amenorrhœa relieved promptly by apioline.

In connection with Dr. Huzza's case Dr. Hutchins mentioned a case of amenorrhœa in a young lady, relieved by cascara sagrada prescribed for constipation.

Dr. Hardon said he had seen very great depression, almost collapse, follow a dose of cascara and consequently had always been afraid of it. He could see how cascara might relieve amenorrhœa as it produced engorgement of the pelvic viscera.

Dr. Baird, Dr. Cooper and Dr. Duncan said they had used it in chronic constipation and in the constipation of pregnancy, and they had never seen any bad effect follow its use.

Dr. Hutchins reported a case of impetigo, or impetigo-contagiosa, which according to Crocker covers both varieties, occurring in a gentleman kindly referred to him by Dr. McRae. Examined first in a poor light, the disease, characterized by yellowish, crusted lesions and some pustules, all discrete, occupying only the bearded portions of face, suggested somewhat a parasitic sycosis, or ringworm of the beard. The patient had a history of ringworm of the neck, and at the edge of one cheek there was a superficial, circumscribed scaly, patch, hair follicles not involved. Hairs were easily extracted from one of the crusted lesions, and microscopic examination showed absence of the fungus. Seen in a better light, the diagnosis of impetigo was made. On the theory of infection with pus-cocci—an ointment in proportion of gr. v hydrarg. bichlor. to ungt. simp ℥i. (made by dissolving the mercury in alcohol qs. and incorporating in the base) was applied and patient ordered to wash the face only in bichloride water. Recovery in two weeks, whereas, a diagnosis of ringworms would have necessitated epilation and the prognosis of a duration of, at least, two or three months. A suppurating furuncle on the back of the neck might have been the source of infection. A case was mentioned which seemed to prove that an ordinary tinea of the skin might invade the face and yet leave the beard unaffected. Under the microscope the fungus of parasitic sycosis seemed larger than that of "tinea corporis."

Dr. McRae reported a case of chromidrosis following Keyes operation for varicocele. The condition was limited to the left side of the scrotum, the side on which the operation was done.

Atlanta Society of Medicine met June 17, with Dr. W. P. Nicolson in the chair.

Dr. W. S. Elkin reported a case of supra-pubic cystotomy for removal of stone. Patient male, age 34 years, gave history of six years' trouble. Came under Dr. Elkin's care a few weeks ago. By sounding the bladder a stone was detected, and operation decided upon. Two weeks ago operation was performed and this evening he presents patient almost well. Technique of operation was as follows: A bag was inserted into the rectum and about a pint of water injected into the bag. The bladder was then dilated with a solution of Boracic acid 10 grs. to ℥i. Incision was then made in median line from about 2 inches below umbilicus extending 2 inches towards the pubes. A stone weighing 500 grs. was removed, bladder was thoroughly washed, one or two stitches put in upper part of wound and the rest packed with antiseptic dressings, and wound allowed to heal by granulation. For a day or so urine was voided enirely through the incision, but by degrees it was partly voided by the urethra, and now the bladder empties itself almost entirely through the urethra. At this time wound is almost healed and it is dressed externally with the usual antiseptic dressing.

Dr. Hardon reported a case of obstruction of the cystic duct successfully treated by cholecystotomy. The patient, a woman 28 years of age, the mother of five children, had always been well until about seven years ago, when she began to suffer with attacks of "bilious colic." In the intervals between the attacks her health was good. About five years ago she first noticed a lump in the right side below the ribs, which gradually increased in size to the present time. Her general health began to fail four years ago, and weakness and emaciation increased until she was confined to the bed nearly all the time. The appetite remained fair, the bowels were constipated. There was no jaundice at any time. She had no pain except during the paroxysms of colic.

On examination, an elastic, fluctuating tumor could be felt on the right side, commencing at the lower border of the liver and extending obliquely downward and to the left as far as the umbilicus. It was apparently about five inches long and two inches broad. The lower extremity could be pushed upward and to the left in a circle, of which the upper extremity of the tumor was the axis. It was apparently attached to the liver by its upper extremity. It was tender to the touch.

A diagnosis was made of distention of the gall bladder from obstruction, and it was decided to perform cholecystotomy with a view to its relief. An incision was made in the median line, extending two inches above the navel and one inch below it. The distended gall bladder at once came into view. It was punctured with a trocar and a half pint of clear serum evacuated. The opening was then enlarged with scissors, and a finger introduced, which came into contact with a mass of gallstones of various sizes, which were removed to the number of thirty-eight. The finger was then passed down to the orifice of the cystic duct, and a stone found lying impacted in the duct with a firm constriction between it and the gall bladder. This stone could not be removed by manipulation, and therefore a bistoury was passed down to the constriction, and its edges slightly nicked at various points. By further effort the stone was then dislodged and removed through the gall bladder. Examination of the cystic and common ducts showed that no other obstruction was present. The incision in the gall bladder was closed by a continued Lembert suture of catgut, and dropped back into the abdomen. The incision in the abdominal wall was closed and dressed in the usual manner.

The patient made a good recovery without a single unfavorable symptom. Her highest temperature was 100.5, on the evening of the second day, and fell to normal on the fourth day. It is now the eleventh day and convalescence is established.

In this operation valuable assistance was rendered by Drs. Rosser and Gwin, of Conyers, and Dr. Gibson, of Newton Co., and the rapid recovery of the patient was largely due to the faithful manner in which the after treatment was carried out by the latter gentleman.

Dr. Gaston said, while the common method was to attach the bladder to the abdominal wall, he approved the course taken by Dr. Hardon, there being nothing calling for the common procedure. Said dilatation of the cystic duct for removal of the impacted stone might have been preferable to nicking. Said Tait had performed the operation oftener perhaps than any one else, his operations numbering in the neighborhood of thirty.

Dr. J. B. Baird reported a case of threatened abortion from retroversion. Patient was three months pregnant; she was suffering from frequent pains and hemorrhage. Examination showed marked retroversion of the fundus, the cervix being impacted under the pubes. Placing patient in knee-chest position, reduction was easily accomplished, patient experiencing immediate sense of relief. Viburnum prunifolium was ordered. There has been no repetition of the trouble.

Dr. M. B. Hutchins reported a case of scleroderma. Will be published in August number.

Dr. J. G. Earnest reported case of laparotomy. Patient negro woman. Examination revealed a tumor of some size in the abdomen. Uterus measured about five inches. Patient was subject to sinking spells, at the time of which she would be cold and pulseless at the wrist. An operation was decided on. Incision revealed a hard glistening tumor, cutting like parchment; deep down there was some fluid. It had a pedicle of considerable size under and to the right of the umbilicus. The growth was so extensive and so adherent to intestines, etc., that it was considered unwise to attempt removal. Abdomen was closed and dressed antiseptically. Temperature following day was 103. It yielded readily to quinine. Patient got along pretty comfortably for a week, when examination revealed fæcal odor about the wound showing there had been ulceration of the intestine and escape of fæces. The abdomen was re-opened, ulcerations found and sutured ; the cavity was carefully washed out and the abdomen closed. The patient fell into other hands, but there were subsequent ulcerations and finally the patient died, but no autopsy was allowed. The points of interest are the nature and source of the tumor.

Dr. W. S. Elkin reported a case of laparotomy performed that day on a woman, removing two cystic tumors, exhibiting specimens.

